# Elixir of Bark and Iron

**Published:** 1862-06

**Authors:** 


ELIXIR OF BARK AND IRON.—We have received from O. H. P. Champlin, Druggist and Apothecary, 319 Main Street, a bottle of *Elixir Peruvian Bark and Protoxide of Iron.* The following is the formula for its preparation:

| | | |
|---|---|---|
| ℞ | Solution Pritoxide Ferri, - - - - | ʒvi |
| | Ext. Cort. Calisaya (Cinchonia et Quinia,) - - | ʒv |
| | Spiritus Vini Recti (80 per cent) - - - - | ʒxiv |
| | Syrupus Simplex, - - - - - - | ʒxx |
| | Tinctura Cort. Auranti, - - - - - | ʒi |
| | Tinctura Cort, Limonio, - - - - - | ʒi |
| | Tinctura Cort. Cinamomi, - - - - - | ʒi |
| | Tinctura Caryophylli, - - - - - | vi gtts. |

"A peculiarity of this combination consists in presenting a Protosalt of Iron, with the active principles of the best variety of Peruvian Bark, in the from of a pleasant Elixir or Cordial."

"As Protoxide of Iron is much more readily assimilable than any other form of the metal, a less amount is required as a dose. The Elixir of Bark and Iron contains in each tablespoonful about three grains of Iron, and what is equivalent to one-third of a grain each of Cinchonine and Quinine. This amount, or half a wine-glassful, may be regarded as a medium dose for adults, two or three times a day."

We have prescribed this newly introduced compound of Bark and Iron since receiving the specimen bottle, and all our patients speak very highly of the agreeable taste as compared with other preparations of Bark. We regard it as the most pleasant, and at the same time, efficient and reliable compound of Bark and Iron, yet introduced to the profession. We understand that all physicians will be gladly furnished with specimen bottles, gratuitously, upon application to Mr. Champlin, who is agent for the manufucturers, James R. Nichols & Co., Boston,

